# Ring‐shaped plaque on the face

**DOI:** 10.1002/ski2.272

**Published:** 2023-07-26

**Authors:** Stella Riemann, Ewan A. Langan, Patrick Terheyden, Katharina Boch

**Affiliations:** ^1^ Department of Dermatology University of Lübeck Lübeck Germany; ^2^ Dermatological Sciences University of Manchester Manchester UK

## Abstract

Granulomatosis Miescher is a very rare, chronic, granulomatous (non‐necrobiotic) disease. Clinical characteristics are a slowly centrifugally growing brownish plaques with partly yellowish parts and telangiectasias. Histology shows perivascular epithelioid cell granulomas with few giant cells and only isolated necrobiosis lesions.

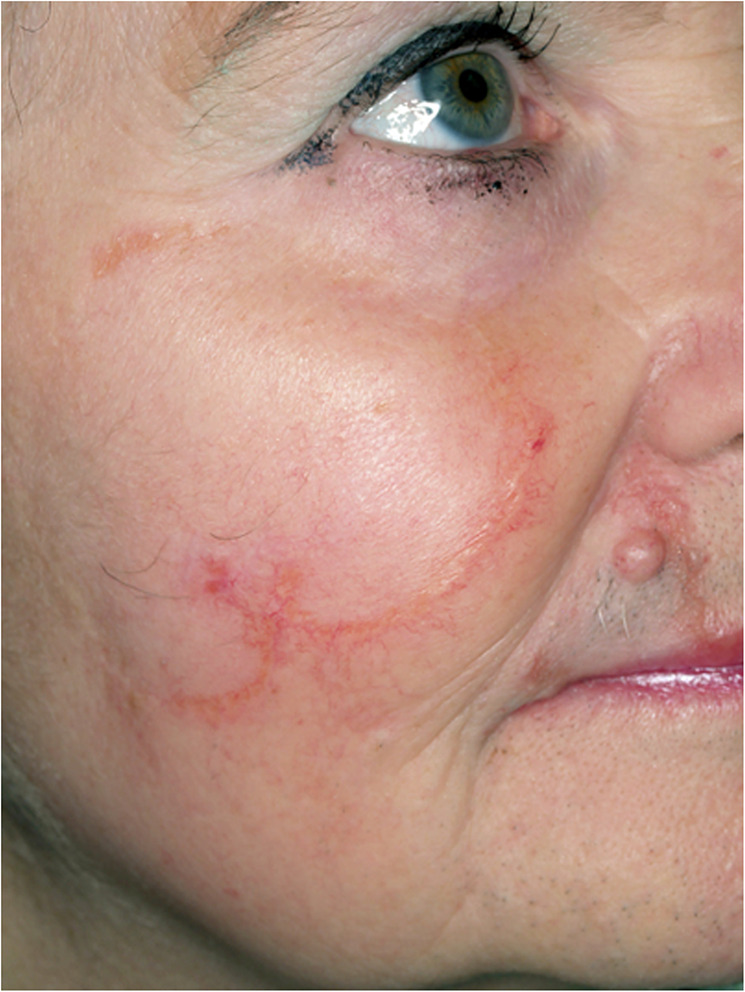

A 53‐year‐old female presented with a ring‐shaped central atrophic plaque on the right cheek and upper lip (Figure 1a), with slightly elevated yellow coloured borders and telangiectasia. The lesion was present for several years and showed a centrifugal growth pattern. The remaining integument was unremarkable. There was a past medical history of mild hypertension only. Due to the clinical picture the main differential diagnoses were cutaneous lupus erythematosus or granulomatous skin disorder, for example, cutaneous sarcoidosis, granuloma annulare, actinic granuloma, necrobiosis lipoidica and cutaneous tuberculosis. The full blood count, C‐reactive protein, erythrocyte sedimentation rate and immunofixation were within normal limits. Antinuclear antibodies, soluble interleukin‐2 receptor, and haemoglobin A1c levels were unremarkable. Mycobacterium tuberculosis infection was excluded by polymerase chain reaction and QuantiFERON‐TB testing. Histopathologic findings showed subepidermal non‐caseating granulomas without necrobiosis and isolated giant cells (Figure 1b). Miescher's granuloma was diagnosed.^1^, ^2^ Miescher's granuloma is part of the necrobiosis lipoidica disease spectrum. Interestingly, diabetes is less frequently associated with Miescher's granuloma.^1^ The histological hallmarks also differ slightly. Besides destruction of the elastic network and minimal changes in the collagen fibres, more multinucleate giant cells and epithelioid cells and less necrobiosis is seen in Miescher's granuloma.^2^ The cause of Miescher's granuloma is unknown.

**FIGURE 1:**
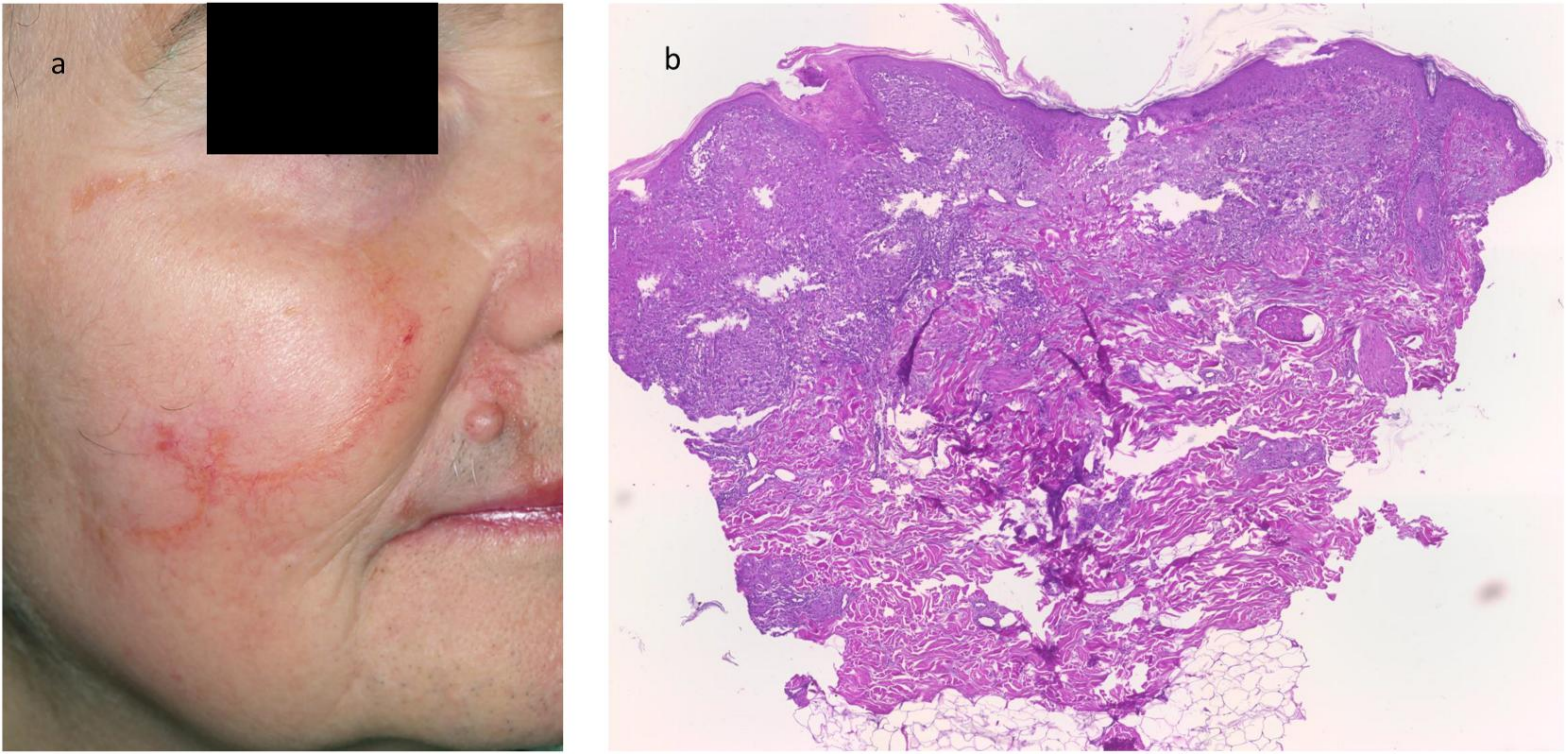
Clinical and histological findings. (a) Ring‐shaped plaque with slightly elevated borders ridge with telangiectasias on the right cheek and upper lip. (b) Granulomatous inflammation pattern with no necrobiosis and a multinucleated giant cell reaction (H&E staining, 10x).

## CONFLICT OF INTEREST STATEMENT

The authors declare no conflicts of interest.

## AUTHOR CONTRIBUTIONS

**Stella Riemann:** Conceptualisation; writing – original draft. **Ewan A. Langan:** Writing – review and editing. **Patrick Terheyden:** Writing – review and editing. **Katharina Boch:** Supervision; visualisation; writing – original draft.

## ETHICS STATEMENT

Not applicable.

## Data Availability

The data that support the findings of this study are available on request from the corresponding author. The data are not publicly available due to privacy or ethical restrictions.

## References

[1] Mehregan AH , Altman J . Miescher's granuloma of the face. A variant of the necrobiosis lipoidica‐granuloma annulare spectrum. Arch Dermatol. 1973;107(1):62–64. 10.1001/archderm.107.1.62 4682543

[2] Tronnier M , Mitteldorf C . Histologic features of granulomatous skin diseases. Part 1: non‐infectious granulomatous disorders. J Dtsch Dermatol Ges. 2015;13(3):211–216. 10.1111/ddg.12610 25721629

